# Creative illustration for choosing-wisely recommendations on asymptomatic bacteriuria of the Network Young Infection Medicine e.V. – jUNITE

**DOI:** 10.1007/s15010-024-02434-3

**Published:** 2025-03-19

**Authors:** Kristin Heenemann, Sarah Kotsias-Konopelska, Jutta Pikalo, Sophie Schneitler, Viktoria Schneitler, Anna Schwabe, jUNITE Study group

**Affiliations:** 1 Institute of Virology, Center for Infectious Diseases, Faculty of Veterinary Medicine, University of Leipzig, Leipzig, Germany; 2 Center for Global Health, Charité Universitätsmedizin Berlin, Berlin, Germany; 3 Institute for Parasitology, University of Veterinary Medicine, Vienna, Austria; 4 Institute of Medical Microbiology and Hygiene, Saarland University, Homburg, Saar Germany; 5 Institute for Medical Microbiology and Hospital Hygiene, University Hospital Duesseldorf, Duesseldorf, Germany; 6 Institute for Hygiene and Public Health, Bonn University Hospital, Bonn, Germany

**Keywords:** Bacteriuria, Urinary tract infection, Antibiotic stewardship, Network young infection medicine, jUNITE

## Abstract

Description of the pictures

These three pictures illustrate the ‘*Choosing-wisely recommendations: Patients with asymptomatic bacteriuria should not be treated with antibiotics*.’ They were designed for the creative competition for the “German Society for Infectious Diseases” in 2023 and were awarded second place.

The core message of the recommendations was presented in various ways with these images. In Fig. 1 an owl is used as a symbol of wisdom. An angel and a devil as an expression of the “good” versus “bad” are used in the 2nd picture. The 3rd figure illustrates a sporting scene where the goal can be reached when making the correct decision.
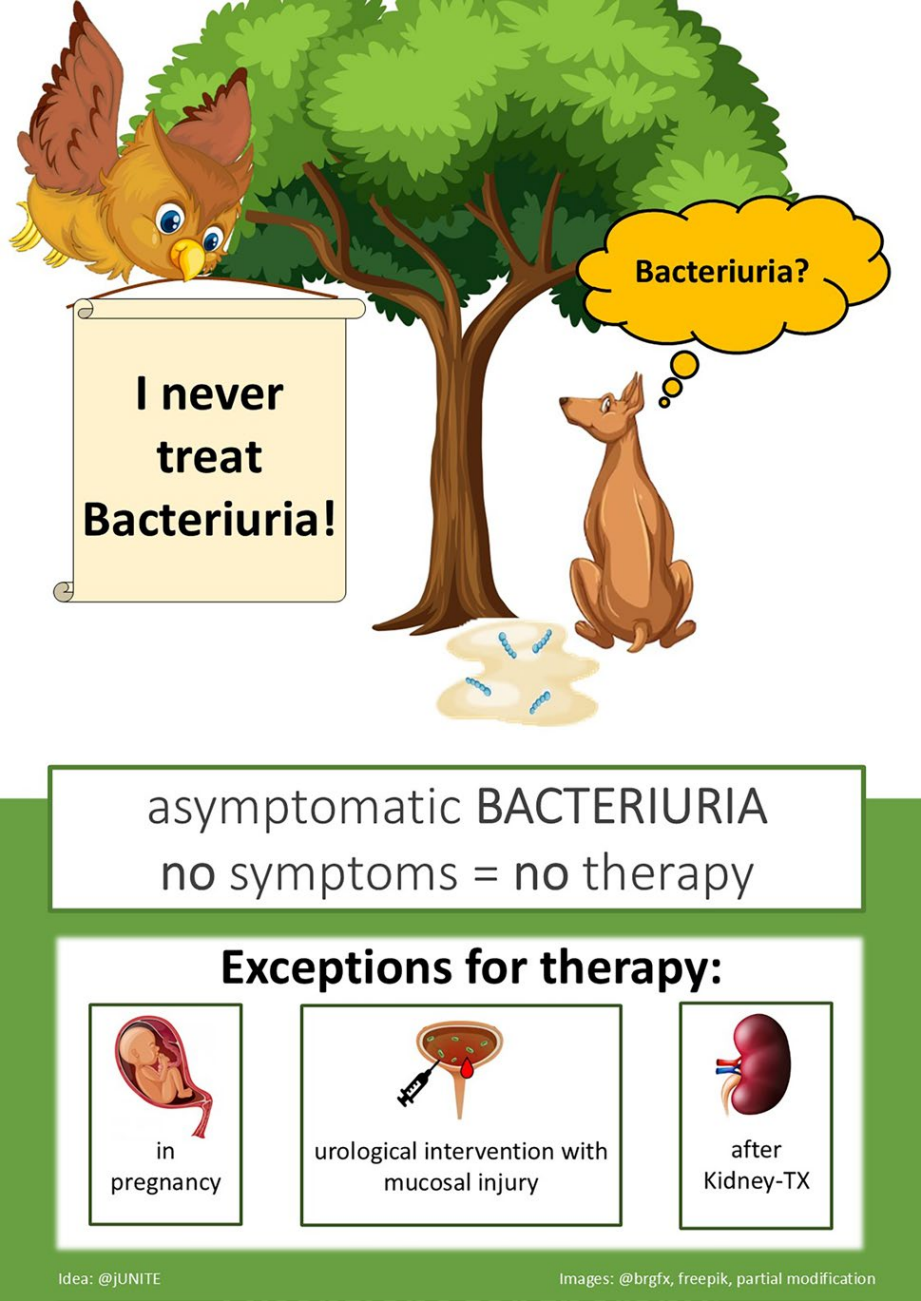



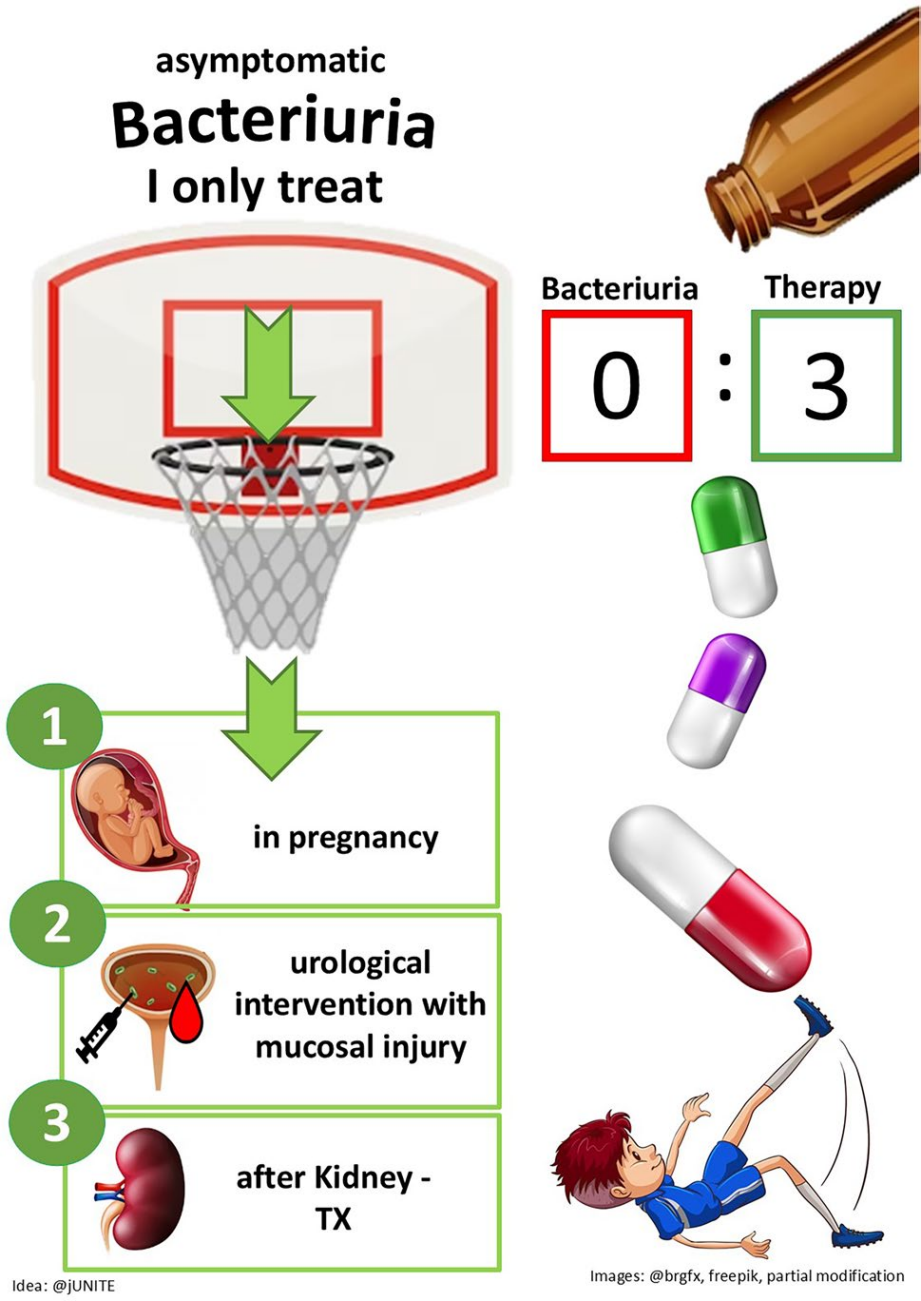



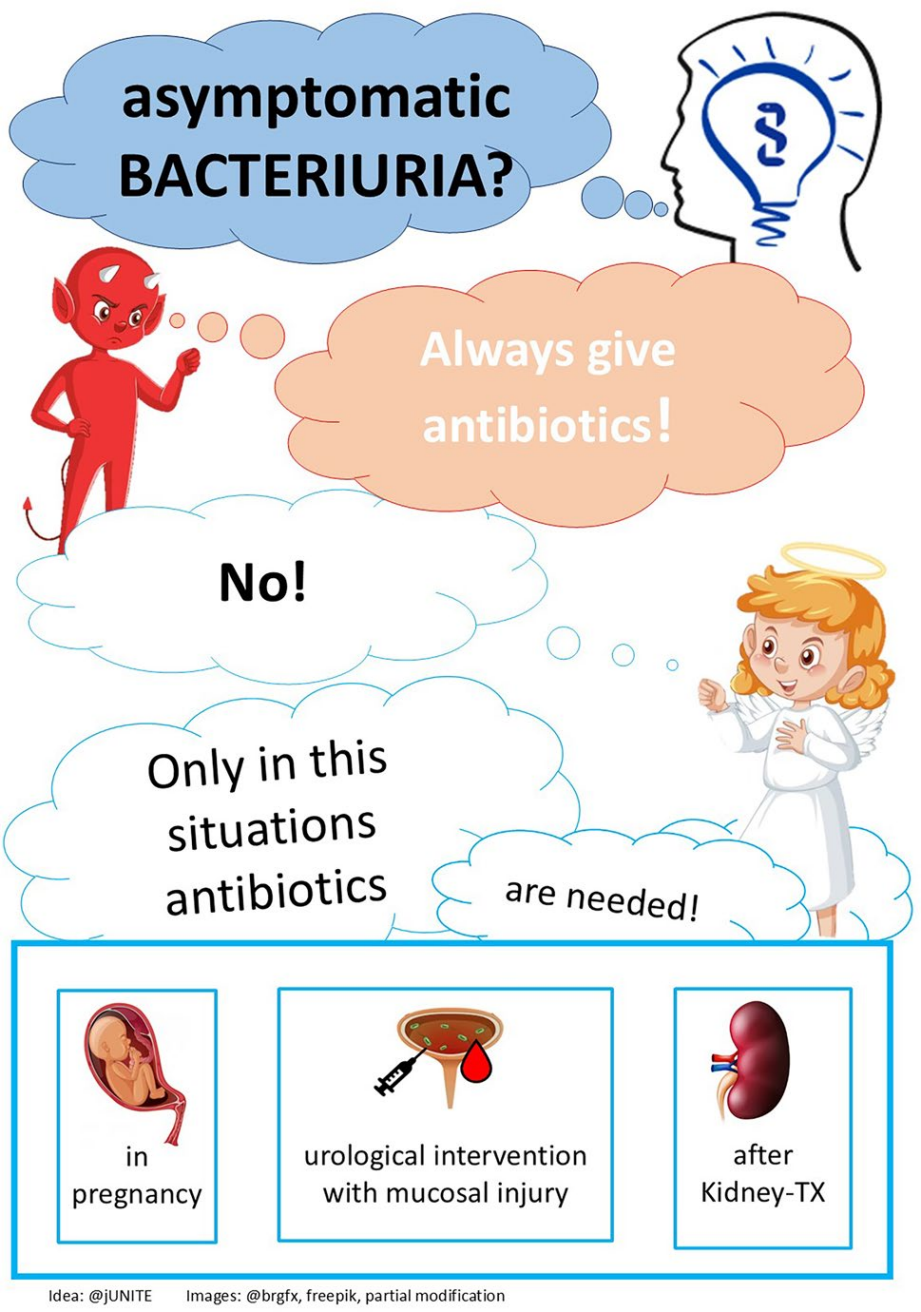


For this purpose, a comic illustration has been chosen in an adapted style, integrating elements of humour to increase memorability.

The pictures were modified according to freepik (@brgfx www.freepik.com).

## Data Availability

No datasets were generated or analysed during the current study.

